# Using tree-based methods for detection of gene–gene interactions in the presence of a polygenic signal: simulation study with application to educational attainment in the Generation Scotland Cohort Study

**DOI:** 10.1093/bioinformatics/bty462

**Published:** 2018-06-19

**Authors:** Joeri J Meijsen, Alexandros Rammos, Archie Campbell, Caroline Hayward, David J Porteous, Ian J Deary, Riccardo E Marioni, Kristin K Nicodemus

**Affiliations:** 1 Centre for Genomic and Experimental Medicine, Institute of Genetics and Molecular Medicine, University of Edinburgh, Edinburgh, UK; 2 Centre for Cognitive Ageing and Cognitive Epidemiology, University of Edinburgh, Edinburgh, UK; 3 Department of Genetics, Smurfit Institute of Genetics and Institute of Neuroscience, Trinity College Dublin, Dublin, Ireland; 4 MRC Human Genetics Unit, Institute of Genetics and Molecular Medicine, University of Edinburgh, Edinburgh, UK; 5 Department of Psychology, University of Edinburgh, Edinburgh, UK

## Abstract

**Motivation:**

The genomic architecture of human complex diseases is thought to be attributable to single markers, polygenic components and epistatic components. No study has examined the ability of tree-based methods to detect epistasis in the presence of a polygenic signal. We sought to apply decision tree-based methods, C5.0 and logic regression, to detect epistasis under several simulated conditions, varying strength of interaction and linkage disequilibrium (LD) structure. We then applied the same methods to the phenotype of educational attainment in a large population cohort.

**Results:**

LD pruning improved the power and reduced the type I error. C5.0 had a conservative type I error rate whereas logic regression had a type I error rate that exceeded 5%. Despite the more conservative type I error, C5.0 was observed to have higher power than logic regression across several conditions. In the presence of a polygenic signal, power was generally reduced. Applying both methods on educational attainment in a large population cohort yielded numerous interacting SNPs; notably a SNP in *RCAN3* which is associated with reading and spelling and a SNP in *NPAS3*, a neurodevelopmental gene.

**Availability and implementation:**

All methods used are implemented and freely available in R.

**Supplementary information:**

Supplementary data are available at *Bioinformatics* online.

## 1 Introduction

Historically, genomic association studies have focused almost exclusively on single-loci and/or polygenic risk score (PGRS) associations. These methods have been very successful; however, frequently they do not explain the total genetic variance of a trait estimated by twin studies. Therefore, it is also important to consider non-additive genetic effects such as epistasis in the complex genetic architecture of human traits. Epistasis has been described as one genetic locus masking or modifying alleles of other loci (Bateson and Mendel, 1909) or a deviation from additivity of two genetic variants on a phenotypic trait (Fisher, 1919). Epistasis, in the sense of ‘deviation from additivity’ can be defined as either antagonistic (a model where the interaction decreases or blocks the effect of each individual allele) or synergistic (where a combination of alleles exacerbates the effect of each allele individually). Many—if not most—complex traits might have different components of genomic architecture of varying importance—e.g. strongly associated single SNPs, a polygenic component and an epistatic component. The evaluation of statistical learning methodologies for the detection of these different components, to our knowledge, has not been performed.

Even though epistasis has been observed and is well documented in multiple non-human organisms (Brockmann *et al.*, 2000; Cheng *et al.*, 2011; Grice *et al.*, 2015; He *et al.*, 2016; Huang *et al.*, 2012; Mackay, 2014), whether or not epistasis exists and plays a vital role in human traits remains an open debate (Hill *et al.*, 2008; Huang and Mackay, 2016; Mackay and Moore, 2014; Sackton and Hartl, 2016; Webber, 2017). According to the ‘omnigenic’ model, in complex traits the disease-related genetic signal tends to be spread across the genome, resulting in genes without direct statistical association to the trait. Therefore, the ‘omnigenic’ model states that, due to a large interconnection between gene regulatory networks, most heritability can be explained by the surrounding genes outside the core disease-related genes, which likely includes epistasis (Boyle *et al.*, 2017). In general, human epistatic studies have shown limited success, partially due to the use of restrictive methods such as searching within subsets of loci or for specific SNP interaction sizes (e.g. hypothesis-driven analysis) in order to lower the number of tests that need to be performed and thus the resulting statistical correction that has to be applied.

Recently, increasing efforts have been placed on addressing the statistical and computational problems related to the detection of epistasis in large datasets. Machine learning (ML) algorithms are increasingly used to ascertain classifiers for either data reduction or feature selection. These include tree-based methods like random forest (RF), classification and regression trees (CART) (Chen *et al.*, 2011; García-Magariños *et al.*, 2009; Stephan *et al.*, 2015) and likelihood ratio Mann-Whitney tests (Lu *et al.*, 2012). García-Magariños *et al.* (2009) simulated genotype data containing interacting SNPs under multiple scenarios (sample size, missing data, minor allele frequencies and several penetrance models). This study found that CART and RF were equally good in detecting interacting SNPs. Even though the study simulated 99 different scenarios with 100 replicates each, the simulated datasets are very small (two ‘causal’ SNPs plus 98 null SNPs) and do not reflect the scale or complexity of modern genomic studies.

**Fig. 1.**
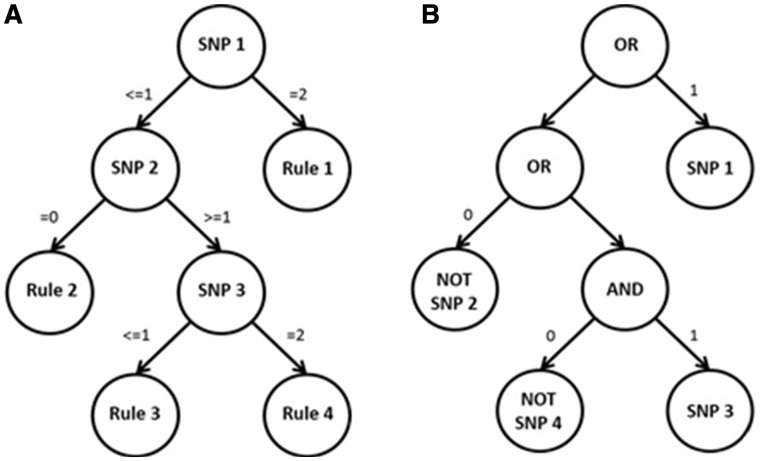
Visual representation of a C5.0 and logic tree. (**A**) C5.0 decision tree; (**B**) logic tree

We sought to apply greedy non-parametric decision tree-based methods—C5.0 and logic regression—for the detection of epistasis in large-scale studies, as these methods explicitly model interactions. C5.0 constructs rule-based decision trees using solely the Boolean operator OR (Fig. 1A) whereas logic regression allows for Boolean operators AND, OR and NOT (Fig. 1B). Note that logic regression is a regression framework therefore allowing for the construction of multiple trees (e.g. multiple trees acting as predictors in a regression model), whereas C5.0 constructs multiple rulesets and is not embedded in a regression framework. To date, C5.0 has never been applied to genetic data in the search for interactions, whereas logic regression has been shown to be effective in detecting main effects and interactions in genetic data and could be used as a comparison method (Chen *et al.*, 2011; Kooperberg *et al.*, 2001; Ruczinski *et al.*, 2003, 2004; Schwender and Ickstadt, 2008).

We sought a complex, but well studied trait to test these approaches. Educational attainment (EA) is a highly heritable complex trait (Calvin *et al.*, 2012; Krapohl *et al.*, 2014) and is highly influenced by social and other environmental factors; however, SNP-based heritability estimates that genetic factors contribute to around 20% of variation across individuals, while average twin-based heritability is around 40% (Rietveld *et al.*, 2013). The largest GWAS to date investigating years of education as a proxy of EA observed 74 statistically significant SNPs (Okbay *et al.*, 2016) of which 72 were replicated in the same study using the large UK Biobank cohort. PGRS derived from the same GWAS explained 3.9% of the variance in years of education in an independent sample. This large gap of missing heritability (Δ$h_{\text{twin}}^{2}$–$h_{\text{SNP}}^{2}$) is in similar to that found in other complex traits, however the moderate correlation with traits showing evidence of epistatic contribution e.g. personality traits (Jang *et al.*, 1996; Loehlin *et al.*, 2003; de Moor *et al.*, 2012; Power and Pluess, 2015; Vukasović and Bratko, 2015) hints towards an epistatic contribution.

In this study, we applied C5.0 and logic regression on simulated epistatic data under multiple scenarios to show their capability of detecting interacting loci in a large genetic study. We sought to assess the performance of C5.0 and logic regression to detect epistatic components alone, plus in the presence of a polygenic signal in order to inform about the methodological development of models that include effects of single SNPs, additive or polygenic components as well as epistasis. To our knowledge, this will be the first simulation study to date to examine the detection of epistasis in the presence of a strong polygenic signal. We applied both methods on the genome-wide SNP data from the Generation Scotland: the Scottish Family health Study (GS: SFHS) cohort to investigate whether there is evidence for an epistatic contribution to years of education as a measurement of educational attainment.

## 2 Materials and methods

### 2.1 Statistical methodology

#### 2.1.1 Classification and regression trees (CART)

CARTs are decision tree-based methods that can be interpreted as a set of decisions leading along a path to a final prediction. CART methods utilize classifiers (measurements) to ‘split’ the data into partitions. CART methods solely use the Boolean operator OR to split a classifier (e.g. male OR female). CART methods grow a tree by including classifiers (recursive partitioning), calculating for every split the ‘impurity’ or misclassification rate, and define a split with the lowest impurity. Commonly-used impurity measurements are the Gini index for classification-based methods and sum of squared residuals for regression-based methods. CART methods keep recursively partitioning the dataset until no split that decreases impurity can be made or when the size of the terminal nodes (e.g. subjects in node) is less than some user-defined value or is 1. This most often leads to a large tree where some terminal nodes only contain a small number of individuals. The complexity of a tree can be decreased by pruning sections of the tree that provide little power to classify observations.

#### 2.1.2 C5.0 and logic regression

C5.0 is a modified version of Quinlan’s non-parametric C4.5 classification algorithm (Quinlan, 1992). C5.0 builds decision trees, performs rule-based models and evaluation of variable importance (Kuhn and Johnson, 2013; Wu *et al.*, 2008). C5.0 decision trees are built by using information entropy (1).

(1) $$\inf o^{s}{}_{before}=-\sum_{i=1}^{m}p_{i}\log p_{i}$$

where *p_i_* is the probability of a given class *i* as the outcome for each of *m* possible classes and *S* is the split.

To build a tree containing optimal splits, C5.0 assesses, for each node, the normalized information gain which acts as the purity criterion. For each node C5.0 calculates the information entropy before (1) and after (2) a split.

(2) $$\inf o^{s}{}_{after}=-\sum_{i=1}^{k}\inf o_{i}\frac{n_{i}}{n}$$

where *S* is the split; *K* is the number of partitions; *n_i_* is the number of samples *i* assigned to partition *K*; *n* is the total number of samples and *info_i_* is the sum of the information entropy in the *i*th resulting partition.

For a given node with split *S* and *K* partitions, C5.0 calculates the information entropy for each resulting partition. This is subsequently multiplied by the proportion of samples assigned to that partition (*n_i_*/*n*). This adds a weight to each partition, which is summed over all partitions resulting in the information entropy after split *S*. A lower information entropy after the split implies an information gain (positive difference) and therefore a decrease in uncertainty. If entropy increases (negative difference) C5.0 stops adding splits. The information gain is normalized to allow for the consideration of each class. C5.0 then selects the class with the highest normalized information gain. This process is repeated recursively for smaller subsets.

Each top-to-bottom path in the final tree is collapsed into a so-called *rule*. C5.0 evaluates each rule on independent conditional statements, thereby assessing whether or not they can be generalized by removing terms in the conditional statement. This process is called rule-based pessimistic pruning and in short removes branches that are not contributing to the improvement of the trees classification. As a final step, C5.0 assigns each rule to a class by calling a vote. The class with the highest vote is used. Results in a single pruned tree where each possible combination from the top node to bottom node in the tree is a so-called ruleset.

Logic regression is a non-parametric adaptive regression method (Ruczinski *et al.*, 2003). Logic regression is largely based on the same principles as a CART, but in contrast to CART, logic regression constructs logic trees (*L*). Logic trees are Boolean combinations (AND, OR and NOT) of binary predictors e.g. *L*_1_ = SNP_3_ or [SNP_1_ and (not SNP_4_ and not SNP_2_)]. This increases the complexity compared to CART which solely applies the Boolean operator OR. A logic tree can be used as a predictor in a regression model (3). Due to its adaptive nature, logic regression estimates the coefficients (*β*s) and Boolean expressions (*L*s) at the same time.

(3) $$y=\beta_{0}+\beta_{1}L_{1}+\beta_{2}L_{2}+\cdots +\beta_{p}L_{p}$$

By doing so, logic regression tries to minimize the scoring function associated with a model type (e.g. residual sum of squares for quantitative outcomes). For the construction of logic trees, logic regression starts at a random starting point and applies a greedy hill climbing algorithm, which keeps adding predictors to the model as long as the misclassification rate goes down and only stops when the misclassification rate goes up.

### 2.2 Simulation and genetic methodology

#### 2.2.1 Generation Scotland

Generation Scotland: the Scottish Family Health Study (GS: SFHS) is a large, family-based cohort study sampled from the general population in Scotland (www.generationscotland.org). The study design has been widely documented (Smith *et al.*, 2006, 2013). In short, 24 000 individuals were recruited in the study during a five-year period (2006–2011). The individuals were deeply phenotyped for a wide variety of traits such as lifestyle factors, family history and health outcomes. DNA of 20 128 GS: SFHS individuals were analyzed by means of high density genome wide bead array genotyping (Illumina OmniExpress 700K SNP GWAS and 250K exome chip). DNA results of 134 individuals were excluded during quality control leaving 19 994 genotyped individuals.

We removed single nucleotide polymorphisms (SNPs) and individuals >5% missing data and removed SNPs with a minor allele frequency <1%. We used Genome-wide Complex Trait Analysis (GCTA) (Yang *et al.*, 2011) to extract a list of genetically-unrelated individuals, giving a total of 7372 individuals (relatedness < 0.025, corresponding to second degree cousins). For the simulation study, we selected 5000 individuals at random from the unrelated set.

We selected the gene-rich chromosome 19 (10 756 SNPs) for analysis. Using PLINK (Purcell *et al.*, 2007) we performed linkage disequilibrium (LD) pruning on chromosome 19 (window size = 50 kb, step size = 5 kb and r^2^ threshold = 0.1), leaving 1705 SNPs in linkage equilibrium (LE). From the LD pruned dataset we designated the potential set of ‘causal’ SNPs in a minor allele frequency range of 0.4–0.5 (584 SNPs). From this pool, high minor allele frequency SNPs were selected to ensure equally high levels of statistical power across all simulations. All analyses were performed twice: once on the linkage disequilibrium pruned (1705 SNPs) and again on the unpruned (10 756 SNPs) chromosome 19 datasets.

#### 2.2.2 Simulation of phenotypes

We simulated phenotypes under the alternative hypothesis (H_1_) and null hypothesis (H_0_) with 500 replicates per condition. All added errors (ɛ) were drawn from a standard normal distribution N (μ = 0, σ^2^ = 1). To ensure unbiased simulation, bias calculations were performed to assess possible over/under estimations of coefficients. Coverage was calculated to assess the probability that the sum of the estimated coefficients fell in the 95% confidence interval using a regression model.

*Polygenic phenotype*

We selected 200 SNPs from the potentially causal SNP pool to form a polygenic phenotype. In this model each SNP explains the same amount of variation (R^2^ = 1.5 × 10^−3^) with a total R^2^ of 0.3 (30%) (4). Simulations were performed using the Linkage-Disequilibrium Adjusted Kinships (LDAK) software (Speed *et al.*, 2012). Bias was observed at 0.18 and coverage was 96%. The bias calculation in the polygenic phenotype is larger compared to other phenotypes.

(4) $$y=\beta_{1}{\text{SNP}}_{1}+\beta_{2}{\text{SNP}}_{2}+\beta_{3}{\text{SNP}}_{3}\ldots +\beta_{2\mathrm{00}}{\text{SNP}}_{2\mathrm{00}}+\varepsilon$$

*2-SNP interacting phenotypes*

Two SNPs not used for simulating the polygenic phenotype were selected at random from the potentially causal SNP pool. We simulated 2-SNP interacting phenotypes assuming each individual SNP has a small but present main effect (β_1_ ≠ 0 and β_2_ ≠ 0) (5).

(5) $$y=\beta_{1}{\text{SNP}}_{1}+\beta_{2}{\text{SNP}}_{2}+\beta_{3}{\text{SNP}}_{1}*{\text{SNP}}_{2}+\varepsilon$$

We simulated three levels of 2-SNP interactions (weak, intermediate and strong) each explaining a different amount of variation (Table 1). The weak interaction phenotype strength was simulated to represent an interaction that would not be detected by a regression model after adjusting for multiple testing by means of a Bonferroni correction (mean *P*-value = 3.1 × 10^−2^; median *P*-value = 1.8 × 10^−3^). The strong interaction phenotypes had a mean *P*-value = 1.3 × 10^−10^ and median *P*-value = 3.5 × 10^−17^ to assess whether C5.0 and logic regression were capable of detecting a strong signal; this phenotype was used as a proof of principle. Intermediate phenotypes (mean *P*-value = 3.6 × 10^−4^; median *P*-value = 2.0 × 10^−7^) were simulated to fall between the two extremes (Table 1). Bias calculations were all close to 0 (strong = 4.0 × 10^−3^, intermediate = 6.7 × 10^−4^ and weak = −5.0 × 10^−3^) and coverage was 96% for the strong phenotype and 94% for both the intermediate and weak phenotypes.

**Table 1.** Two-SNP interaction models, R^2^ and *P*-values

Model	β_1_,β_2_	β_3_	$R_{2\text{SNPinteraction}}^{2}$ (%)	$R_{\text{fullmodel}}^{2}$ (%)	Mean *p*_interaction_	Median *p*_interaction_
Strong	0.2	0.24	1.6	35.6	1.3 × 10^−10^	3.5 × 10^−17^
Intermediate	0.125	0.15	0.82	17.8	3.6 × 10^−4^	2.0 × 10^−7^
Weak	0.07	0.09	0.35	6.8	3.1 × 10^−2^	1.8 × 10^−3^

*3-SNP interacting phenotypes*

Three SNPs not previously used for simulating the polygenic phenotype were selected at random from the potentially causal SNP pool. We analyzed these phenotypes independently. Three levels (weak, strong and pure) of 3-SNP interactions were simulated including all possible 2-SNP interactions (6).

(6) $$y=\beta_{1}{\text{SNP}}_{1}+\beta_{2}{\text{SNP}}_{2}+\beta_{3}{\text{SNP}}_{3}+\beta_{4}{\text{SNP}}_{1}*{\text{SNP}}_{2}+\beta_{5}{\text{SNP}}_{1}*{\text{SNP}}_{3}+\beta_{6}{\text{SNP}}_{2}*{\text{SNP}}_{3}+\beta_{7}{\text{SNP}}_{1}*{\text{SNP}}_{2}*{\text{SNP}}_{3}+\varepsilon$$

We simulated a weak and strong 3-SNP interacting phenotype explaining a different amount of variation; we set the *β*s of the strong interaction to be twice as large as the weak ones. (Table 2). Also, we simulated a pure 3-SNP interaction where in Equation 6*β*_1_ to *β*_6_ are all set to 0. Bias calculations were again all close to 0 (pure = −7.41 × 10^−4^, strong = 2.26 × 10^−4^ and weak = −5.87 × 10^−4^) and coverage was 97% for the pure phenotype and 93% for both the intermediate and weak phenotypes.

**Table 2.** Three-SNP interaction models, R^2^ and *P*-values

Model	β_1_,β_2,_β_3_	β_4,_ β_5,_β_6_	β_7_	$R_{2\text{SNPinteraction}}^{2}$ (%)	$R_{3\text{SNPinteraction}}^{2}$ (%)	$R_{\text{fullmodel}}^{2}$ (%)	Mean *p*_interaction_	Median *p*_interaction_
Pure	0	0	0.4	0.04	1.86	30.1	1.0 × 10^−14^	1.1 × 10^−22^
Strong	0.05	0.1	0.2	0.19	0.41	39.9	4.5 × 10^−4^	6.6 × 10^−7^
Weak	0.025	0.05	0.1	0.15	0.1	14.3	7.7 × 10^−2^	1.3 × 10^−2^

*Combined polygenic and interacting phenotypes*

To assess the capability of C5.0 and logic regression to detect gene-gene interactions even in the presence of an additive or polygenic component we simulated an interaction in the data used for the polygenic simulations, using SNPs not included in the polygenic component.

*Null phenotype*

To assess the type I error, we modelled a phenotype under H_0_ where all *β*s are set to 0; therefore, y = ɛ. Bias was observed as 1.2 × 10^−3^ with a coverage of 94%.

*Main effect*

To rule out the possibility that the power was driven by the larger *β*s of the main effects within the interaction phenotypes, we simulated 500 replicates that only included the main effect of the strong phenotype; y = *β*_1_SNP_1_ + *β*_2_SNP_2_ + ε (where *β*_1_ and *β*_2_ = 0.2). As this interaction has the largest coefficients we chose this setting as a proof of principle for all other phenotypes with smaller coefficients. Bias was observed as 9.0 × 10^−3^ and coverage of 96.4%. The strong two-SNP main effect signal was then combined with the 200-SNP polygenic signal.

#### 2.2.3 Data pre-processing and parameter settings

Genotype data were converted to ordered vectors. Logic regression only allows for binary predictors; therefore, we dichotomised the genotype data into dominant and recessive predictors, i.e. genotype {0, 1, 2} becomes dominant {0, 1, 1} and recessive {0, 0, 1}. Missing genotypes were imputed by means of median imputation before analysis.

#### 2.2.4 Calculating type I error and power

We defined the type I error for C5.0 as the percentage of trees constructed under H_0_. Power was defined as the percentage of constructed sets of rulesets under H_1_ containing all of the simulated interacting SNPs.

**Fig. 2.**
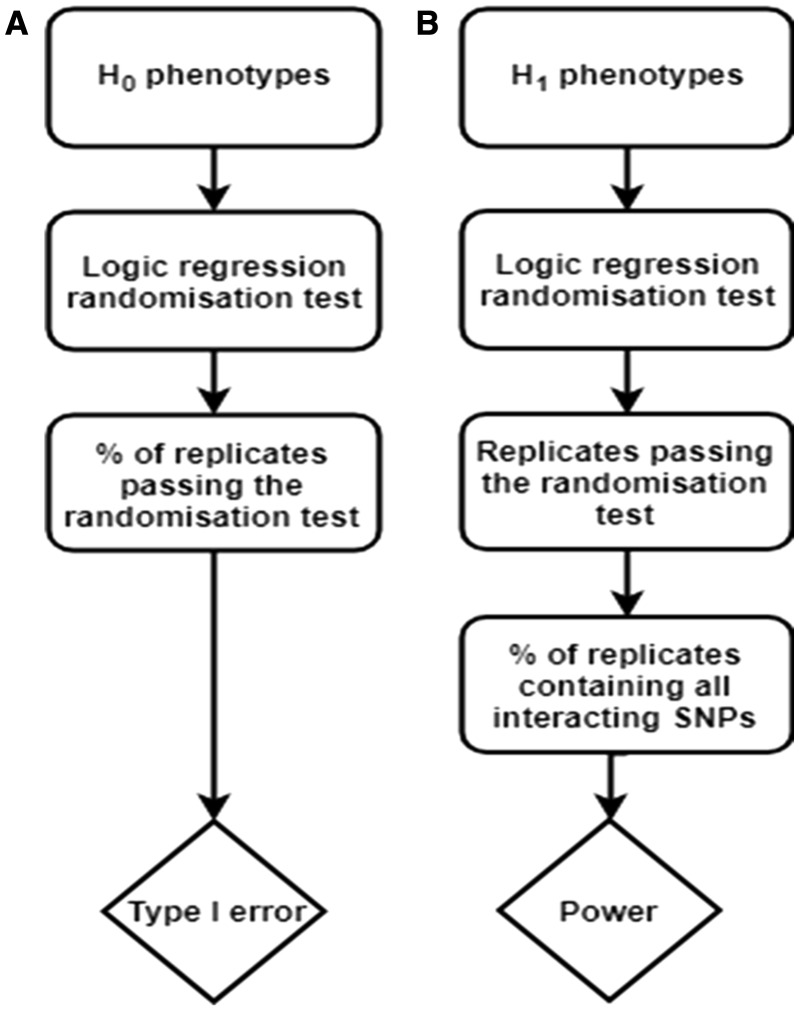
Flow chart for logic regression analyses

For logic regression, we assessed the presence of a signal under H_0_ and H_1_ by performing a randomisation test. Each replicate was permuted 100 times; the number of instances the original model had a lower score (residual sum of squares) than 95% of permuted models (α = 0.05) was derived. For type I error, we counted the number of times that the replicate passed the randomisation test when no signal was present and divided by 500. For power, we considered only those replicates that passed the randomisation test, and similarly for the calculation for type I error (Fig. 2A). Then we assessed if the logic trees contained all the simulated interacting SNPs. If the replicate (a) passed the randomisation test and (b) the simulated interacting SNPs were present, this was considered as a ‘true positive’ and we summed the number of these replicates and divided by 500 to obtain power (Fig. 2B).

## 3 Results

### 3.1 Type I error

We observed that C5.0 has a type I error of 0% when using the LD pruned data and 0.6% when using the LD unpruned data. Using the randomisation test we observed that logic regression has a 5.8% type I error using LD pruned data, rising to 6.4% when using LD unpruned data.

### 3.2 Power

#### 3.2.1 LD-pruned data

Power results for pruned and unpruned data for both methods can be found in Table 3. C5.0 detected rulesets in 11.4% polygenic replicates with the LD pruned dataset. Of these, 79% were based on a single SNP, 19% on two SNPs and 2% on four SNPs. All observed SNPs in the rulesets were from the 200 SNPs used to create the polygenic phenotypes, with no un-associated SNPs in any rulesets. C5.0 detected the two interacting SNPs in 100% of the strong replicates. This number decreased to 99.2% in the intermediate and 8.6% in the weak replicates (Supplementary Table S1). No rulesets were created that included other non-interacting SNPs; in other words, no false-positive SNPs were included in any of the rulesets generated for the interaction simulation replicates. Furthermore, in 35% of the weak replicates no ruleset was created; the remainder contained just one of the two interacting SNPs. In the combined polygenic and 2-SNP interaction phenotype analysis, C5.0 shows that it is capable of distinguishing additivity from interactions by detecting the two interacting SNPs in 100% of strong and 23% of intermediate replicates (Supplementary Table S1). In the combined polygenic and weak interaction analyses C5.0 did not detect a single ruleset in 98.8% of the replicates. In the remaining 1.2%, C5.0 was not able to detect both interacting SNPs (Supplementary Table S1). Higher order interactions, i.e. 3-SNP interaction, were also detected using C5.0. We observed a power of 100, 100 and 90.4% of all three interacting SNPs in the pure, strong and weak 3-SNP interaction phenotypes (Supplementary Table S2). When combining these phenotypes with a polygenic signal, the interaction power remained 100% for the pure and strong phenotypes and dropped to 11.2% for the weak phenotype (Supplementary Table S2). For replicates that only contained two SNPs with main effects and no interaction, we observed that C5.0 detected 62.6% rulesets containing solely one of the two main effect SNPs, in 35.2% both SNPs and in 2.2% no rulesets. When combined with a polygenic signal this dropped to 0.4% for both SNPs and one of the main effect SNPs in 16% while it did not detect any ruleset in the remaining 84%.

**Table 3.** Power of C5.0 and logic regression in pruned and unpruned data, with and without polygenic component

Condition	C5.0: − polygenic	LR: − polygenic	C5.0 + polygenic	LR + polygenic
2-SNP, Pruned, Weak	8.6%	77.0%	0%	9.6%
2-SNP, Pruned, Intermediate	99.2%	98.8%	23%	82.8%
2-SNP, Pruned, Strong	100%	99.8%	100%	98.8%
2-SNP, Unpruned, Weak	19.8%	0.3%	0.6%	0%
2-SNP, Unpruned, Intermediate	98.2%	17.6%	41.6%	2.2%
2-SNP, Unpruned, Strong	100%	52.8%	100%	23.4%
3-SNP, Pruned, Weak	90.4%	89.0%	3.6%	53.6%
3-SNP, Pruned, Strong	100%	99.6%	100%	95.2%
3-SNP, Pruned, Pure	100%	99.6%	100%	98.4%
3-SNP, Unpruned, Weak	91.0%	14.7%	24.0%	2.2%
3-SNP, Unpruned, Strong	100%	35.4%	100%	21.0%
3-SNP, Unpruned, Pure	100%	50.2%	100%	35.2%

LR, Logic Regression.

Randomisation test-based analyses showed the power of logic regression using the LD pruned data ranged between 89.6% (combined polygenic and weak 3-SNP interaction) and 100% (Table 4) for phenotypes containing interactions and 99.6% for the polygenic-only phenotype. For each of the 500 polygenic replicates logic regression created a model containing eight SNPs. These models contained either 2 (1%), 3 (2.6%), 4 (11%), 5 (24.7%), 6 (31.1%), 7(22.9%) or 8 (6.6%) polygenic SNPs. This means that, in all replicates minus the 6.6% containing 8 polygenic SNPs, logic regression includes several SNPs that can be defined as false-positives when a polygenic signal is present because these SNPs have been LD-pruned; thus, their presence is not due to correlation with a polygenic SNP.

**Table 4.** Power of logic regression based on randomization tests

Model	Power: Pruned	Power: Unpruned
Weak 2-SNP interaction	97.4	64.6
Intermediate 2-SNP interaction	100	100
Strong 2-SNP interaction	100	100
30% Polygenic + Weak 2-SNP interaction	89.6	54
30% Polygenic + Inter. 2-SNP interaction	100	89.6
30% Polygenic + Strong 2-SNP interaction	100	100
Weak 3-SNP interaction	100	99.2
Strong 3-SNP interaction	100	100
Pure 3-SNP interaction	100	100
30% Polygenic + Weak 3-SNP interaction	100	92.6
Polygenic + Strong 3-SNP interaction	100	100
Polygenic + Pure 3-SNP interaction	100	100
Polygenic model	99.6	94.4

For all 2-SNP interaction analyses logic regression created trees containing eight SNPs with the exception of ten trees (0.15%) containing 1 (1 tree), 3 (1 tree), 4 (2 trees), 5 (2 trees), 6 (1 tree) or 7 (3 trees) SNPs. For the strong and intermediate 2-SNP phenotypes, logic regression created in 99.8 and 98.8% replicates logic trees containing both interacting SNPs (Supplementary Table S3). Several of the remaining SNPs in these trees were false positives, not due to LD. This dropped to 77% in the weak 2-SNP phenotype, with 1% containing no interacting SNPs. When combining the polygenic and epistatic phenotypes the trees contained the interacting SNPS in the strong (98.8%), intermediate (82.8%) and weak (9.6%) phenotypes (Supplementary Table S3). Furthermore, 45.3% of the created combined polygenic-weak trees contained no interacting SNPs. The majority of trees in the higher order 3-SNP interaction analyses contained the interacting SNPs (pure 99.6%, strong 99.6% and weak 89%; Supplementary Table S4a). This number decreased when adding the polygenic component (pure 98.4%, strong 95.2% and weak 53.6%; Supplementary Table S4b). No trees were observed containing solely non-interacting SNPs.

#### 3.2.2 Unpruned data

C5.0 detection of rulesets in the polygenic model increased to 53.4% when not LD pruned (Supplementary Table S5). Seventy-six point eight percent of observed SNPs in the rulesets were used to create the 200 SNP polygenic phenotypes or were in LD with a polygenic SNP (r^2^ > 0.25). Compared to the pruned set analyses, the percentage accurately detecting 2-SNP interactions by C5.0 remained 100% for the strong phenotype but decreased to 98.2% in the intermediate phenotype. The percentage accurately detected 2-SNP interactions increased to 19.8% in the weak phenotype (Supplementary Table S6). However, it has to be noted that C5.0 detected in 3.2% (16 rulesets) non-interacting random SNPs of which 12 contained SNPs in LD with the true signal (r^2^ > 0.25; this threshold was set to be consistent with the value for LD pruning). In the combined polygenic and interaction phenotype analysis, the power remained unchanged for the strong phenotype. The power was again higher in the intermediate (41.6%) and weak (0.6%) phenotype, but 10.6 and 6.6%, of replicates respectively, contained at least one false positive SNP (Supplementary Table S6), which could be linked to LD structure. We observed no change in C5.0 interaction power in the pure and strong three 3-SNP interaction phenotypes (100%) and an increase to 91% in the weak phenotype (Supplementary Table S7). Only the weak interaction phenotype showed a higher power compared to the pruned analysis of 24% (Supplementary Table S7).

Randomisation test-based analyses using the non-LD pruned data showed six interaction phenotypes having a lower power when using non-LD pruned data compared to LD pruned data. Power dropped to 94.4% for the polygenic phenotype. The largest differences were observed with the combined polygenic and 3-SNP interaction phenotype (35.6%) and weak 2-SNP phenotype (32.8%) (Table 3). When analyzing the polygenic phenotype we observed that logic regression created 15.4% trees containing no polygenic SNPs. This dropped to 1.4% when taking LD structure into account (r^2^ > 0.25). The rest of the trees contained either 1 (33.4%), 2 (29.6%), 3 (14.6%), 4 (5.6%), 5 (1%) or 6 (0.4%) polygenic SNPS and in 89.3% in combination with numerous SNPs in LD with the polygenic SNPs. The power of logic regression for the two interacting SNP phenotypes was 52.8% in the strong, 17.6% in the intermediate and 0.3% in the weak phenotype (Supplementary Table S8). This dropped further in the combined analysis to 23.4% in the strong, 2.2% in the intermediate and 0% in the weak phenotype (Supplementary Table S8).

We observed that in the higher order phenotypes, the trees contain three forms of the interacting SNPs is 50.2% (pure), 35.4% (strong) and 14.7% (weak) (Supplementary Table S9a). In line with previous observed results when adding the polygenic signal, the numbers again lowered to 35.2% (pure), 21% (strong) and 2.2% (weak) (Supplementary Table S9b). For all phenotypes a percentage of trees were created containing non interacting SNPs; however, the majority of these trees contained SNPs in LD (r^2^ > 0.25) with the interacting SNPs (for a detailed outline see Supplementary Table S10).

### 3.3 Application to educational attainment in GS: SFHS

Having assessed our methods by simulation, we wished to test the approach on a large set of complex trait data. We extracted 7012 unrelated GS: SFHS individuals of which 6765 individuals had a measure of years of education, measured by ordered categories (e.g. 0: 0 years, 1: 1–4 years, 2: 5–9 years). We performed a linear regression analysis between years of education controlling for sex and age, and extracted the residuals to act as an adjusted years of education measurement (Zhao et al., 2012). Finally, we applied C5.0 and logic regression on the residual years of education outcome using 131 821 whole genome SNPs in LE (LD pruning settings; window size = 50 kb, step size = 5 kb and r^2^ threshold = 0.1). C5.0 detected 32 rulesets associated with educational attainment containing in total 30 SNPs (Supplementary Table S11). The logic regression model did not pass the randomisation test (α = 0.05) so will not be discussed further.

## 4 Conclusions and discussion

When using LD-pruned genetic data we observed that C5.0 is capable of distinguishing additivity from interactions. C5.0 created rulesets based on a polygenic phenotype in 11.4% of the replicates; however, the majority of these (78.9%) were based on one single polygenic SNP. C5.0 correctly detected both interacting SNPs in 100 and 99.2% in the strong and intermediate phenotypes. Even though the interaction strength was low, C5.0 was capable of detecting the signal in 8.6% of the weak 2-SNP interaction replicates, of which none would be significant using a standard regression model after adjusting for multiple testing. For the 3-SNP (higher order) interaction phenotype, C5.0 was able to detect all three SNPs in 100% of the pure and strong and in 90.4% of the weak phenotype. When combining the polygenic and interaction phenotypes C5.0 was able to distinguish the interaction signal from the polygenic signal in 100 and 23% of the strong and intermediate 2-SNP phenotypes. For the weak phenotype C5.0, was not able to detect any ruleset in 98.8% of the replicates showing it to be protective against spurious results when the interaction term is of low magnitude. Similar results were observed in the 3-SNP combined analyses. As no rulesets were observed under H_0_, we conclude that C5.0 had a low type I error. We could not see any evidence that our previously observed results were driven by main effects when analyzing strong main effect data only. This indicates that C5.0 is detecting rulesets based on conditional dependencies and not on large main effects. We observed that LD structure has an impact on the performance of C5.0. In all but four phenotypes that include an interaction component the amount of accurately detected interactions decreased using unpruned data (Table 4).

We observed that logic regression is capable of accurately detecting all interacting SNPs in all but one phenotype either combined with additivity and using LD pruned or unpruned data. Logic regression was not capable of detecting both interacting SNPs in the 2-SNP interaction including a polygenic signal in the LD unpruned phenotype. However, we observed a slightly inflated type I error (5.8 and 6.8%), which is in line with the developers’ statement that logic regression is likely to overfit (Kooperberg *et al.*, 2001). It should be noted that logic regression has a high overall power when performing a randomisation analysis, however when looking into the SNPs used to create the initial model, logic regression-built trees using random SNPs therefore the overall randomisation test-based power is high but the frequency of inclusion of spurious SNPs in a model is also high. Furthermore, as mentioned logic regression applies a greedy hill climbing algorithm. Greedy hill climbing algorithms stop when the last predictor included does not improve the prediction rate. As logic regression applies a random starting point, it risks creating a set of Boolean combinations of binary predictors that may reflect a local optimum rather than the global optimum. One solution to circumvent this issue is to apply a global optimum search technique e.g. simulated annealing.

We observed 32 rulesets containing 30 putative epistatic SNPs associated with educational attainment (EA) in Generation Scotland. From the thirty SNPs, 18 could be mapped to genes, two were in genes previously associated with mental health or cognitive performance (rs196433, chr1, *RCAN3* and rs17100828, chr14, *NPAS3*). *RCAN3* is associated with reading and spelling (Luciano *et al.*, 2013) while *NPAS3* acts as a master regulator of neuropsychiatric risk genes (Michaelson *et al.*, 2017). Of the remaining 16 genes, none showed a clear association with any phenotype. We sought to investigate whether these SNPs have been previously reported in the large EA GWAS study which observed 74 statistically significant SNPs (Okbay *et al.*, 2016). None of the SNPs observed in this study overlapped or could be considered a proxy SNP (r^2^ > 0.8) with the previously reported GWAS results. One explanation for the lack of overlap might be because GWAS searches for single SNPs associated with a phenotype while C5.0 searches for conditional dependencies associated with the phenotype. Therefore, one could say that both methods search for different pieces of the same puzzle. The results strengthen the assumption that interacting SNPs play an important role in educational attainment (Supplementary Table S12).

The main strength of this study is that we assessed the capability of both C5.0 and logic regression in detecting simulated genetic interactions under a wide range of settings including a strong polygenic signal. We suggest that C5.0 rulesets might be used as predictors within a regression model alongside single SNPs and additive or polygenic components (Nicodemus *et al.*, 2014). The same can be done with logic trees. Limitations lie in the modest sample size (N = 5000) and the use of only causal SNPs with a large MAF (0.4–0.5). We did not simulate phenotypes containing multiple SNP interactions (polygenic-epistatic phenotype) which is biologically plausible.

In conclusion, we have shown that C5.0 and logic regression are capable of detecting simulated genetic interactions in a wide range of association levels and even in the presence of a strong polygenic component. We showed that when applying both methods LD pruning helps by improving the power and reducing the type I error. Finally, using C5.0 we were able to detect 32 rulesets containing 30 SNPs not previously reported with EA in Generation Scotland; *RCAN3* has been previously observed in association with learning and reading while *NPAS3* is involved in neurodevelopment. These methods are capable of detecting SNPs not directly associated to the trait but rather in sets of SNPs that together affect the trait. These methods are well-adapted to testing hypotheses regarding the ‘omnigenic’ model.

## Supplementary Material

Supplementary Tables
