# Supplementary material for: Using tree-based methods for detection of gene–gene interactions in the presence of a polygenic signal: simulation study with application to educational attainment in the Generation Scotland Cohort Study
Source: Bioinformatics. 2018 Jun 19;35(2):181–8. doi: 10.1093/bioinformatics/bty462 (PMC6330004; doi:10.1093/bioinformatics/bty462)
Supplement: Supplementary Tables [file bty462_supplementary_tables.docx]

**Supplementary Table S1. C5.0 results in 2-SNP interaction phenotypes LD pruned data**

| **Phenotypic model** | **%** | **No rulesets** | **Two interacting SNPs** | **One interacting SNP** | **Non-interacting SNPs** |
| --- | --- | --- | --- | --- | --- |
| **Strong 2-SNP interaction** | 100 |  | **✓** |  |  |
| **Intermediate 2-SNP interaction** | 99.2 |  | **✓** |  |  |
|  | 0.8 |  |  | **✓** |  |
| **Weak 2-SNP interaction** | 8.6 |  | **✓** |  |  |
|  | 56.4 |  |  | **✓** |  |
|  | 35 | **✓** |  |  |  |
| **30% polygenic**  **+**  **strong 2-SNP interaction** | 99.6 |  | **✓** |  |  |
|  | 0.4 |  | **✓** |  | **✓** |
| **30% polygenic**  **+**  **Intermediate 2-SNP interaction** | 66 |  |  | **✓** |  |
|  | 0.2 |  |  | **✓** | **✓** |
|  | 23 |  | **✓** |  |  |
|  | 10.8 | **✓** |  |  |  |
| **30% polygenic**  **+**  **Weak 2-SNP interaction** | 0.8 |  |  | **✓** |  |
|  | 0.2 |  |  |  | **✓** |
|  | 0.2 |  |  | **✓** | **✓** |
|  | 98.8 | **✓** |  |  |  |

**Supplementary Table S2. C5.0 results in 3-SNP interaction phenotypes LD pruned data**

| **Phenotypic model** | **%** | **No**  **rulesets** | **Three interacting SNPs** | **Two interacting**  **SNPs** | **One interacting**  **SNP** | **Non-interacting**  **SNPs** |
| --- | --- | --- | --- | --- | --- | --- |
| **Pure 3-SNP interaction** | 99.6 |  | **✓** |  |  |  |
|  | 0.4 |  | **✓** |  |  | **✓** |
| **Strong 3-SNP interaction** | 100 |  | **✓** |  |  |  |
| **Weak3-SNP interaction** | 0.2 |  |  |  | **✓** |  |
|  | 9.4 |  |  | **✓** |  |  |
|  | 90.2 |  | **✓** |  |  |  |
|  | 0.2 |  | **✓** |  |  | **✓** |
| **30% polygenic**  **+**  **pure 3-SNP interaction** | 98.4 |  | **✓** |  |  |  |
|  | 1.6 |  | **✓** |  |  | **✓** |
| **Polygenic**  **+**  **strong 3-SNP interaction** | 98.2 |  | **✓** |  |  |  |
|  | 1.8 |  | **✓** |  |  | **✓** |
| **Polygenic**  **+**  **Weak 3-SNP interaction** | 10.8 | **✓** |  |  |  |  |
|  | 39.4 |  |  |  | **✓** |  |
|  | 0.2 |  |  |  | **✓** | **✓** |
|  | 0.2 |  |  | **✓** |  | **✓** |
|  | 38.2 |  |  | **✓** |  |  |
|  | 10.6 |  | **✓** |  |  |  |
|  | 0.6 |  | **✓** |  |  | **✓** |

**Supplementary Table S3. Logic regression results in 2-SNP interaction phenotypes LD pruned data**

|  | **Without polygenic phenotype** | | | **Combined with polygenic phenotype** | | |
| --- | --- | --- | --- | --- | --- | --- |
| **Phenotypic model** | **%**  **SNP_1_** | **%**  **SNP_2_** | **%**  **SNP_1_ SNP_2_** | **%**  **SNP_1_** | **%**  **SNP_2_** | **%**  **SNP_1_ SNP_2_** |
| **Weak 2-SNP interaction** | 14 | 8 | 77 | 35.3 | 10.3 | 9.6 |
| **Intermediate 2-SNP interaction** | 1 | 0.2 | 98.8 | 15.4 | 1.8 | 82.8 |
| **Strong 2-SNP interaction** | 0.2 | 0 | 99.8 | 1.2 | 0 | 98.8 |

**Supplementary Table S4a. Logic regression results in 3-SNP interaction phenotypes LD pruned data**

|  | **Without polygenic phenotype** | | | | | | |
| --- | --- | --- | --- | --- | --- | --- | --- |
| **Phenotypic model** | **%**  **SNP_1_** | **%**  **SNP_2_** | **%**  **SNP_3_** | **%**  **SNP_1_ SNP_2_** | **%**  **SNP_1_ SNP_3_** | **%**  **SNP_2_ SNP_3_** | **%**  **SNP_1_ SNP_2_ SNP_3_** |
| **Weak 3-SNP interaction** | 0 | 0 | 0 | 2.2 | 2.6 | 6.2 | 89 |
| **Strong 3-SNP interaction** | 0 | 0 | 0 | 0 | 0 | 0.4 | 99.6 |
| **Pure 3-SNP interaction** | 0 | 0 | 0 | 0.2 | 0 | 0.2 | 99.6 |

**Supplementary S4b. Logic regression results in 3-SNP interaction phenotypes LD pruned data**

|  | **Combined with polygenic phenotype** | | | | | | |
| --- | --- | --- | --- | --- | --- | --- | --- |
| **Phenotypic model** | **%**  **SNP_1_** | **%**  **SNP_2_** | **%**  **SNP_3_** | **%**  **SNP_1_ SNP_2_** | **%**  **SNP_1_ SNP_3_** | **%**  **SNP_2_ SNP_3_** | **%**  **SNP_1_ SNP_2_ SNP_3_** |
| **Weak 3-SNP interaction** | 5.6 | 2.8 | 1.6 | 19 | 7.2 | 10.2 | 53.6 |
| **Strong 3-SNP interaction** | 0 | 0 | 0 | 1.2 | 0.8 | 2.8 | 95.2 |
| **Pure 3-SNP interaction** | 0 | 0 | 0 | 0.6 | 0.2 | 0 | 98.4 |

**Supplementary Table S5. Detailed outline of the distribution of decision trees created by C5.0 using the polygenic phenotype**

| **Ruleset size (*n* SNPS)** | **Rounded *%* rulesets**  **(n total = 233)** |
| --- | --- |
| 1 | 39 |
| 2 | 25 |
| 3 | 12 |
| 4 | 9 |
| 5 | 4 |
| 6 | 4 |
| 7 | 2 |
| 8 | 3 |
| 10 | 0.4 |
| 11 | 0.4 |
| 12 | 0.4 |

**Supplementary Table S6. C5.0 results in 2-SNP interaction phenotypes non LD pruned data**

| **Phenotypic model** | **%** | **No**  **rulesets** | **Two interacting**  **SNPs** | **One interacting**  **SNP** | **Non-interacting**  **SNPs** |
| --- | --- | --- | --- | --- | --- |
| **Strong 2-SNP interaction** | 96.8 |  | **✓** |  |  |
|  | 3.2 |  | **✓** |  | **✓** |
| **Intermediate 2-SNP interaction** | 1.8 |  |  | **✓** | **✓** |
|  | 89.4 |  | **✓** |  |  |
|  | 8.8 |  | **✓** |  | **✓** |
| **Weak 2-SNP interaction** | 23.6 | **✓** |  |  |  |
|  | 3.2 |  |  |  | **✓** |
|  | 51.8 |  |  | **✓** |  |
|  | 1.6 |  |  | **✓** | **✓** |
|  | 18.4 |  | **✓** |  |  |
|  | 1.4 |  | **✓** |  | **✓** |
| **30% Polygenic**  **+**  **Strong 2-SNP interaction** | 85.4 |  | **✓** |  |  |
|  | 14.6 |  | **✓** |  | **✓** |
| **30% Polygenic**  **+**  **Intermediate 2-SNP interaction** | 48.6 |  |  | **✓** |  |
|  | 1.8 |  |  |  | **✓** |
|  | 35 |  | **✓** |  |  |
|  | 3.8 |  |  | **✓** | **✓** |
|  | 6.6 |  | **✓** |  | **✓** |
|  | 4.2 | **✓** |  |  |  |
| **30% Polygenic**  **+**  **Weak 2-SNP interaction** | 86.6 | **✓** |  |  |  |
|  | 4.2 |  |  | **✓** |  |
|  | 6 |  |  |  | **✓** |
|  | 0.2 |  | **✓** |  |  |
|  | 2.6 |  |  | **✓** | **✓** |
|  | 0.4 |  | **✓** |  | **✓** |

**Supplementary Table S7 in C5.0 results in 3-SNP interaction phenotypes non LD pruned data**

| **Phenotypic model** | **%** | **No rulesets** | **Three interacting SNPs** | **Two interacting SNPs** | **One interacting SNP** | **Non-interacting SNPs** |
| --- | --- | --- | --- | --- | --- | --- |
| **Pure 3-SNP interaction** | 90 |  | **✓** |  |  |  |
|  | 10 |  | **✓** |  |  | **✓** |
| **Strong 3-SNP interaction** | 88.6 |  | **✓** |  |  |  |
|  | 11.4 |  | **✓** |  |  | **✓** |
| **Weak 3-SNP interaction** | 0.4 |  |  |  | **✓** |  |
|  | 0.2 |  |  |  | **✓** | **✓** |
|  | 4.4 |  |  | **✓** |  |  |
|  | 4 |  |  | **✓** |  | **✓** |
|  | 82.8 |  | **✓** |  |  |  |
|  | 8.2 |  | **✓** |  |  | **✓** |
| **30% Polygenic**  **+**  **pure 3-SNP interaction** | 80.8 |  | **✓** |  |  |  |
|  | 19.2 |  | **✓** |  |  | **✓** |
| **Polygenic**  **+**  **strong 3-SNP interaction** | 82.2 |  | **✓** |  |  |  |
|  | 17.8 |  | **✓** |  |  | **✓** |
| **Polygenic**  **+**  **Weak 3-SNP interaction** | 22.6 |  |  |  | **✓** |  |
|  | 0.4 |  |  |  |  | **✓** |
|  | 39.4 |  |  | **✓** |  |  |
|  | 3 |  |  |  | **✓** | **✓** |
|  | 18.2 |  | **✓** |  |  |  |
|  | 6.2 |  |  | **✓** |  | **✓** |
|  | 5.8 |  | **✓** |  |  | **✓** |
|  | 2.4 | **✓** |  |  |  |  |

**Supplementary Table S8. Logic regression results in 2-SNP interaction phenotypes non LD pruned data**

|  | **Without polygenic phenotype** | | | **Combined with polygenic phenotype** | | |
| --- | --- | --- | --- | --- | --- | --- |
| **Phenotypic model** | **%**  **SNP_1_** | **%**  **SNP_2_** | **%**  **SNP_1_ SNP_2_** | **%**  **SNP_1_** | **%**  **SNP_2_** | **%**  **SNP_1_ SNP_2_** |
| **Weak 2-SNP interaction** | 14.6 | 9.9 | 0.3 | 7.2 | 0.6 | 0 |
| **Intermediate 2-SNP interaction** | 25.6 | 31.8 | 17.6 | 21.4 | 7.4 | 2.2 |
| **Strong 2-SNP interaction** | 21.2 | 22.2 | 52.8 | 33.8 | 23.6 | 23.4 |

**Supplementary Table S9a. Logic regression results in 3-SNP interaction phenotypes non LD pruned data**

|  | **Without polygenic phenotype** | | | | | | |
| --- | --- | --- | --- | --- | --- | --- | --- |
| **Phenotypic model** | **%**  **SNP_1_** | **%**  **SNP_2_** | **%**  **SNP_3_** | **%**  **SNP_1_ SNP_2_** | **%**  **SNP_1_ SNP_3_** | **%**  **SNP_2_ SNP_3_** | **%**  **SNP_1_ SNP_2_ SNP_3_** |
| **Weak 3-SNP interaction** | 19.6 | 4.8 | 11 | 17.1 | 17.7 | 10.3 | 14.7 |
| **Strong 3-SNP interaction** | 4.8 | 1.4 | 9.6 | 5.4 | 34.2 | 9.2 | 35.4 |
| **Pure 3-SNP interaction** | 3 | 0 | 4.4 | 2.4 | 31.4 | 8.6 | 50.2 |

**Supplementary Table S9b. Logic regression results in 3-SNP interaction phenotypes non LD pruned data**

|  | **Combined with polygenic phenotype** | | | | | | |
| --- | --- | --- | --- | --- | --- | --- | --- |
| **Phenotypic model** | **%**  **SNP_1_** | **%**  **SNP_2_** | **%**  **SNP_3_** | **%**  **SNP_1_ SNP_2_** | **%**  **SNP_1_ SNP_3_** | **%**  **SNP_2_ SNP_3_** | **%**  **SNP_1_ SNP_2_ SNP_3_** |
| **Weak 3-SNP interaction** | 20.7 | 11.2 | 13.6 | 8.6 | 6.5 | 3.5 | 2.2 |
| **Strong 3-SNP interaction** | 20.4 | 1.6 | 5.6 | 14 | 29 | 8.4 | 21 |
| **Pure 3-SNP interaction** | 11.8 | 0.8 | 3.8 | 12.4 | 26 | 9 | 35.2 |

**Supplementary Table S10. Percentage of logic trees containing non-interacting SNPs and, of those the percentage in LD with all interacting SNPs**

|  | **% non-interacting SNPs** | **% in LD with all interacting SNPs*** |
| --- | --- | --- |
| **Weak 2-SNP** | 75.2 | 6.8 |
| **Intermediate 2-SNP** | 25 | 20.4 |
| **Strong 2-SNP** | 3.8 | 84.2 |
| **30% Polygenic + Weak 2-SNP** | 91.9 | 0.7 |
| **30% Polygenic + Intermediate 2-SNP** | 69.6 | 10.8 |
| **30% Polygenic + Strong 2-SNP** | 19.2 | 18.3 |
| **Weak 3-SNP** | 4.8 | 0 |
| **Strong 3-SNP** | 0 | 0 |
| **Extreme 3-SNP** | 0 | 0 |
| **30% Polygenic + Weak 3-SNP** | 33 | 0 |
| **30% Polygenic Strong 3-SNP** | 0 | 0 |
| **30% Polygenic Extreme 3-SNP** | 0.8 | 0 |

*this count is the number of trees that contained SNPs that were in LD with all of the SNPs that interacted (e.g., either 2 SNPs or 3 SNPs, depending on the simulation model)

**Supplementary Table S11. C5.0 rulesets associated with educational attainment**

| **Ruleset**  **number** | **Ruleset**  **build** | **Outcome** | **N**  **individuals** | **Ruleset**  **number** | **Ruleset**  **build** | **Outcome** | **N**  **individuals** |
| --- | --- | --- | --- | --- | --- | --- | --- |
| 1 | rs196433 > 0  rs6747637 > 0  rs10125618 = 2  rs7965873 > 0  rs7226712 > 0  rs7256201 = 0 | -0.9582104 | 33 | 17 | rs4416197 = 0  rs6747637 > 0  rs4279287 = 0  rs10216277 > 0  rs7965873 = 0  rs17100828 < 2  rs12923539 < 2 | 0.6721485 | 144 |
| 2 | rs7567614 = 0  rs17057882 < 2  rs10993564 = 2  rs11878345 > 0 | -0.9667753 | 101 | 18 | rs196433 > 0  rs7567614 = 0  rs6747637 > 0  rs10125618 < 2  rs3802609 < 2  rs7965873 > 0  rs1442849 > 0  rs7226712 > 0 | 0.4464759 | 221 |
| 3 | rs6747637 > 0  rs4279287 = 0  rs10216277 = 0  rs4739619 < 2  rs1403257 < 2  rs7965873 = 0  rs12923539 < 2  rs7226712 > 0 | -0.7254973 | 883 | 19 | rs7567614 > 0  rs477995 > 0 | 0.859727 | 212 |
| 4 | rs196433 > 0  rs7567614 = 0  rs6747637 > 0  rs10125618 < 2  rs3802609 < 2  rs7965873 > 0  rs282593 < 2  rs1442849 = 0  rs7226712 > 0 | -0.6437932 | 237 | 20 | rs7567614 = 0  rs6747637 > 0  rs2404867 > 0  rs17057882 < 2  rs10993564 < 2  rs7226712 = 0  rs11878345 > 0 | 0.8822943 | 253 |
| 5 | rs7567614 = 0  rs4416197 = 0  rs6747637 > 0  rs1403257 = 2  rs13334339 > 0  rs12923539 < 2  rs7226712 > 0  rs2206173 < 2 | -0.801764 | 126 | 21 | rs3770613 = 2  rs4416197 = 0  rs7965873 = 0  rs12923539 = 2  rs7226712 > 0 | 1.3422044 | 84 |
| 6 | rs7567614 = 0  rs4416197 > 0  rs6747637 > 0  rs7965873 = 0  rs7226712 > 0 | -0.6803628 | 854 | 22 | rs7567614 = 0  rs4416197 = 0  rs6747637 > 0  rs4739619 = 2  rs7965873 = 0  rs17100828 < 2  rs12923539 < 2  rs7226712 > 0 | 1.04883 | 37 |
| 7 | rs7567614 = 0  rs6747637 = 0  rs10934116 = 0  rs4947631 = 0 | -0.6437932 | 1365 | 23 | rs4416197 = 0  rs6747637 > 0  rs10216277 = 0  rs1403257 = 2  rs7965873 = 0  rs13334339 = 0  rs7226712 > 0  rs2206173 < 2 | 1.4013413 | 33 |
| 8 | rs7567614 = 0  rs10934116 = 0  rs4947631 > 0  rs11054372 = 0 | -0.5760914 | 107 | 24 | rs7567614 = 0  rs4416197 = 0  rs6747637 > 0  rs7965873 = 0  rs12923539 = 2  rs765742 = 1 | 1.4916104 | 73 |
| 9 | rs196433 = 0  rs7567614 = 0  rs6747637 > 0  rs7965873 > 0  rs17100828 < 2  rs7226712 > 0 | -0.5900937 | 219 | 25 | rs196433 > 0  rs6747637 > 0  rs10125618 < 2  rs7965873 > 0  rs282593 = 2 | 2.4690432 | 35 |
| 10 | rs3770613 < 2  rs4416197 = 0  rs6747637 > 0  rs7965873 = 0  rs12923539 = 2  rs7226712 > 0  rs765742 = 0 | -0.4632551 | 167 | 26 | rs7567614 = 0  rs10125618 < 2  rs3802609 = 2  rs7965873 > 0  rs7990443 = 2  rs7226712 > 0 | 1.7468907 | 53 |
| 11 | rs7567614 > 0  rs477995 = 0 | -0.463255 | 161 | 27 | rs6747637 > 0  rs3802609 = 2  rs7965873 > 0  rs282593 < 2  rs7226712 > 0  rs2206173 = 0 | 1.6495813 | 61 |
| 12 | rs7567614 = 0  rs6747637 > 0  rs10125618 < 2  rs3802609 = 2  rs7965873 > 0  rs7990443 < 2  rs282593 < 2  rs2206173 > 0 | -0.2531093 | 167 | 28 | rs4416197 = 0  rs6747637 > 0  rs4279287 = 0  rs10216277 = 0  rs4739619 < 2  rs1403257 = 2  rs7965873 = 0  rs12923539 < 2  rs7226712 > 0  rs2206173 = 2 | 1.3787741 | 30 |
| 13 | rs7567614 = 0  rs6747637 > 0  rs17057882 < 2  rs17100828 < 2  rs7226712 = 0  rs11878345 = 0 | -0.372986 | 727 | 29 | rs6747637 > 0  rs17057882 = 2  rs7226712 = 0 | 1.3562067 | 53 |
| 14 | rs2404867 = 0  rs17057882 < 2  rs10993564 < 2  rs17100828 < 2  rs7226712 = 0  rs11878345 > 0 | -0.0725712 | 779 | 30 | rs17100828 = 2 | 1.5453099 | 24 |
| 15 | rs7567614 = 0  rs6747637 = 0  rs10934116 > 0 | -0.2756765 | 604 | 31 | rs196433 > 0  rs7567614 = 0  rs6747637 > 0  rs10125618 = 2  rs7965873 > 0  rs7226712 > 0  rs7256201 > 0 | 2.0628324 | 45 |
| 16 | rs7567614 = 0  rs4279287 > 0  rs4739619 < 2  rs7965873 = 0  rs7226712 > 0 | 0.4550408 | 297 | 32 | rs7567614 = 0  rs6747637 = 0  rs10934116 = 0  rs4947631 > 0  rs11054372 > 0 | 2.5001753 | 22 |

**Supplementary Table S12: p-values of SNPs observed in C5.0 rulesets in *Okbay et al, 2016*.**

| **MarkerName** | **CHR** | **POS** | **Pval** |
| --- | --- | --- | --- |
| rs196433 | 1 | 24862643 | 0.9484 |
| rs3770613 | 2 | 170146913 | 0.03686 |
| rs4416197 | 2 | 201596926 | 0.3345 |
| rs6747637 | 2 | 212406789 | 0.4191 |
| rs7567614 | 2 | 54386795 | 0.9382 |
| rs10934116 | 3 | 111574943 | 0.1779 |
| rs2404867 | 4 | 136519891 | 0.5269 |
| rs4279287 | 4 | 189151984 | 0.9237 |
| rs17057882 | 5 | 159926960 | 0.04366 |
| rs10216277 | 7 | 4401148 | 0.05367 |
| rs4947631 | 7 | 50603379 | 0.6259 |
| rs4739619 | 8 | 82149338 | 0.2022 |
| rs10125618 | 9 | 6555311 | 0.002604 |
| rs10993564 | 9 | 93272277 | 0.3226 |
| rs3802609 | 10 | 27025659 | 0.6269 |
| rs477995 | 11 | 104737590 | 0.0455 |
| rs1403257 | 11 | 80789029 | 0.5751 |
| rs7965873 | 12 | 44354797 | 0.1481 |
| rs11054372 | 12 | 11757164 | 0.6413 |
| rs7990443 | 13 | 79494019 | 0.584 |
| rs282593 | 13 | 113378882 | 0.5949 |
| rs17100828 | 14 | 33839502 | 0.3818 |
| rs13334339 | 16 | 8615618 | 0.2977 |
| rs12923539 | 16 | 59582084 | 0.4694 |
| rs1442849 | 17 | 8024121 | 0.05291 |
| rs7226712 | 18 | 2874552 | 0.1987 |
| rs765742 | 19 | 57151907 | 0.07446 |
| rs7256201 | 19 | 51705039 | 0.1543 |
| rs11878345 | 19 | 35868773 | 0.7682 |
| rs2206173 | 22 | 35223541 | 0.4449 |
